# Item

**Published:** 1899-09

**Authors:** 


### ITEM.

BRIGHAM HALL, a hospital for the insane, located at Canandaigua, is one of the best institutions of its kind in the country. Dr. D. R. Burrell, the resident physician, is one of the most experienced men in that department of medicine and his assistant, Dr. E. J. Gillette, is a very competent man. Of late the hospital has received voluntary patients. Cases of neurasthenia, and of the alcohol or drug habit, who are not insane, but are willing to conform to the regulations of the hospital, and to the advice of the physicians will continue to be received on voluntarily signing a request for admission.

